# One-pot synthesis of cobalt–rhenium nanoparticles taking the unusual β-Mn type structure†

**DOI:** 10.1039/d0na00097c

**Published:** 2020-04-07

**Authors:** Eirini Zacharaki, G. Marien Bremmer, Ponniah Vajeeston, Maria Kalyva, Helmer Fjellvåg, Patricia J. Kooyman, Anja O. Sjåstad

**Affiliations:** Centre for Materials Science and Nanotechnology, Department of Chemistry, University of Oslo P. O. Box 1033 N-0315 Oslo Norway a.o.sjastad@kjemi.uio.no; Leiden Institute of Physics, Leiden University P. O. Box 9502 2300 RA Leiden The Netherlands; Department of Chemical Engineering, University of Cape Town Private Bag X3 7701 Rondebosch South Africa

## Abstract

Using a facile one-pot colloidal method, it is now possible to obtain monodisperse Co_1−*x*_Re_*x*_ nanoparticles (NPs), with excellent control of Re stoichiometry for *x* < 0.15. Re-incorporation in terms of a solid solution stabilizes the β-Mn polymorph relative to the hcp/ccp variants of cobalt. The NPs are thermally stable up to 300 °C, which may make them attractive as model catalysts.

## Abstract 2

Monodisperse Co_1−*x*_Re*_x_* nanoparticles with the β-Mn structure and excellent control of Re stoichiometry can be obtained for *x* < 0.15, using a facile one-pot colloidal method.

## Graphical abstract

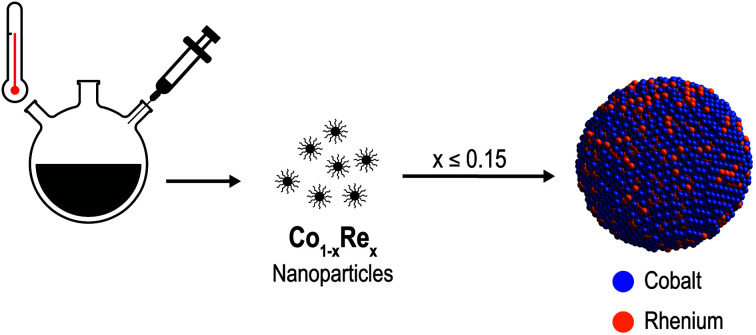

Bimetallic nanoparticles (NPs) offer unexplored opportunities for science and technology, based on size, morphology, and compositional effects.^1^ For catalytic applications, such NPs may exhibit superior performance compared to their monometallic counterparts due to synergy between electronic structure and new types of active sites, induced by the local presence of the second metal.^2,3^ Cobalt–Rhenium (Co–Re) NPs are of direct interest for the Fischer–Tropsch (FT) and ammonia synthesis reactions.^4,5^ Typically, for Co- and Re-promoted Co (Co–Re) catalysts, product selectivity and reaction rate are highly correlated to the metallic particle size, element composition/distribution, polymorphism/stacking disorder, as well as the nature of the support material. In this context, it should be recalled that cobalt may take at least four polymorphic forms; hcp, ccp, bcc, and the primitive cubic β-Mn type structure.^6^ At ambient pressure, metallic cobalt crystallizes as hcp or ccp, where the hcp variant is the thermodynamically stable polytype below 420 °C.^7^ Due to a particle size effect, Co may be stabilized in the ccp form as NPs below 420 °C.^8^ Interestingly, the β-Mn type polymorph is solely observed for Co NPs obtained through colloidal synthesis.^9^ A long lasting debate for FT catalysts concerns the location of the Re promoter in the Co NPs. While some studies indicate that Re forms an alloy within the bulk of the Co NP matrix,^10^ others propose that Re atoms accumulate on the surface of the Co NPs,^11^ or that Re is located just below a surface layer of Co atoms.^12^ However, in these experimental studies,^10,11^ the Co–Re bimetallic catalysts were prepared by impregnation, which gives limited control of particle size and elemental distribution. A more targeted strategy to provide good model materials would be the synthesis of free-standing Co–Re bimetallic nanoparticles *via* colloidal chemistry, with subsequent deposition on a support material followed by a suitable annealing treatment.^13^ To our knowledge, synthetic protocols for colloidal Co–Re NPs are not available. The current study describes for the first time how monodisperse Co–Re NPs can be successfully obtained. Interestingly, using hot injection synthesis, the incorporation of Re as a solid solution stabilizes the β-Mn polymorph. This polymorph has been reported for monometallic Co NPs.^9,14^ Our current diffraction and Density Functional Theory (DFT) modelling results show Re to occupy specific sites in this structure and actually stabilize the β-Mn polymorph relative to the hcp structure.

Uniformly sized Co_1−*x*_Re_*x*_ (*x* < 0.15) NPs are synthesized by thermal decomposition of dicobalt octacarbonyl [Co_2_(CO)_8_; *T*_d_ = 52 °C] and dirhenium decacarbonyl [Re_2_(CO)_10_; *T*_d_ = 173 °C] in *ortho*-dichlorobenzene (*o*-DCB) containing oleic acid (OA). We have modified our previously reported protocol for the synthesis of Co NPs^9*a*^ as follows (Scheme 1): (i) Re_2_(CO)_10_ is introduced to the OA/*o*-DCB solution; (ii) the reaction temperature is increased to 177 °C before the Co_2_(CO)_8_ solution is injected to the system; (iii) the colloidal solution is aged for 2–4 h.

**Scheme 1:**
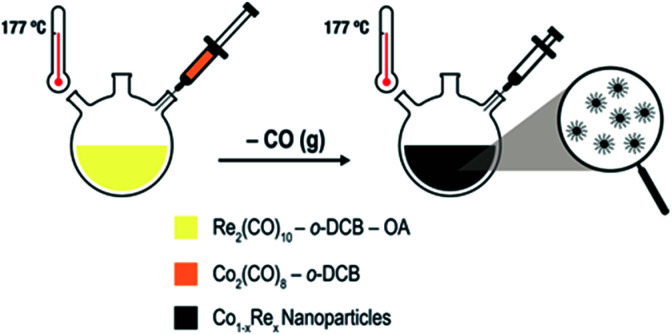
Colloidal Co–Re NPs formed *via* thermal decomposition of carbonyl precursors in the presence of OA and *o*-DCB.

The metal composition of the NPs can be tuned effectively by adjusting the relative amounts of Co- and Re-carbonyl precursors (Tables S1 and S2†). High-resolution transmission electron microscopy – energy dispersive X-ray spectroscopy (HRTEM-EDX) measurements of a number of single Co–Re NPs confirms their bimetallic nature, and average stoichiometries are listed in Table S2 and Fig. S1.†

Fig. 1 shows the synchrotron powder X-ray diffraction (SR-PXRD) pattern of Co–Re NPs with nominal composition Co_0.85_Re_0.15_. Rietveld refinements using the structural model reported by Dinega and Bawendi^9*b*^ confirms the Co–Re NPs have the cubic β-Mn-type structure (space group *P*4_1_32). See Table S3† for refined crystallographic parameters. Notably, the Rietveld fit (Fig. S2†) gives a clear preferential site occupancy for Re-atoms in the 12-fold site. The unit cell expands upon Re incorporation, implying formation of a solid solution. By assuming a linear relationship in the *a*-axis between the two end members Co and Re [*a* = 0.6098(3) nm for Co;^9*a*^ and 0.697(2) nm for Re^15^], the refined value *a* = 0.6199(1) nm for the synthesized Co_0.85_Re_0.15_ NPs corresponds to incorporation of 11.7 at% Re. This value is in good agreement with the Re content (12.2 ± 1.3 at%) determined by HRTEM-EDX (Table S2†).

**Fig. 1:**
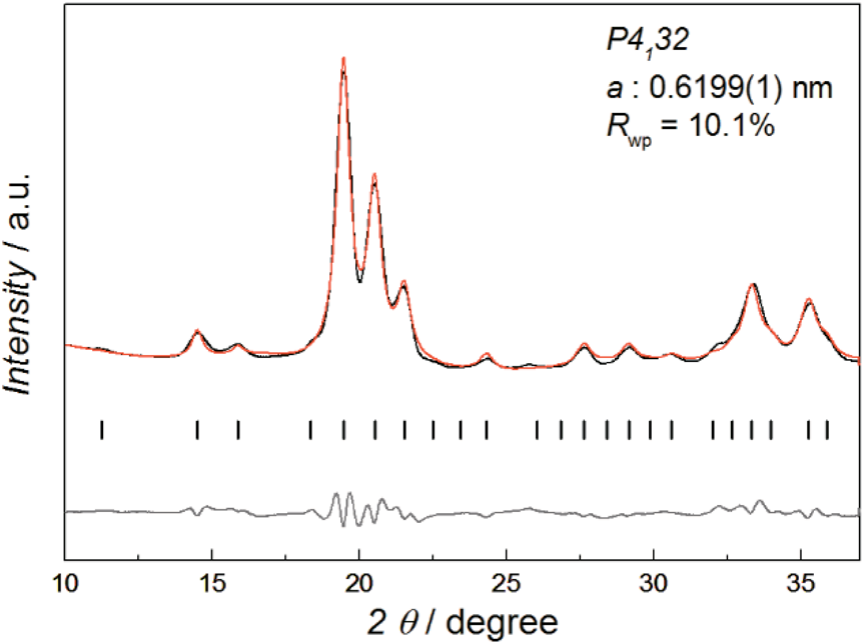
SR-PXRD pattern (black) and Rietveld refinement of Co_0.85_Re_0.15_. The calculated (red) and difference (gray) profiles are shown along with the positions for the Bragg reflections (vertical bars), *λ* = 0.06957 nm.

Fig. 2a shows a representative high-angle annular dark-field scanning transmission electron microscopy (HAADF-STEM) image of NPs with nominal composition Co_0.85_Re_0.15_. The as-prepared particles have a spherical shape with an average diameter of 8 nm and a narrow size distribution (Fig. 2b). HRTEM (Fig. 2c) reveals equidistant lattice fringes and a periodic lattice with no indications of Re segregation. To further document the elemental distribution in the individual NPs, STEM-EDX line scans were recorded (Fig. 2d and e). The compositional line profiles across the two NPs (Fig. 2e) show homogeneous Co and Re distributions, and atomic-level mixing in the Co_0.85_Re_0.15_ crystallites. Based on the SR-PXRD and STEM-EDX results, we conclude that the as-prepared Co_0.85_Re_0.15_ NPs consist of a solid solution.

**Fig. 2:**
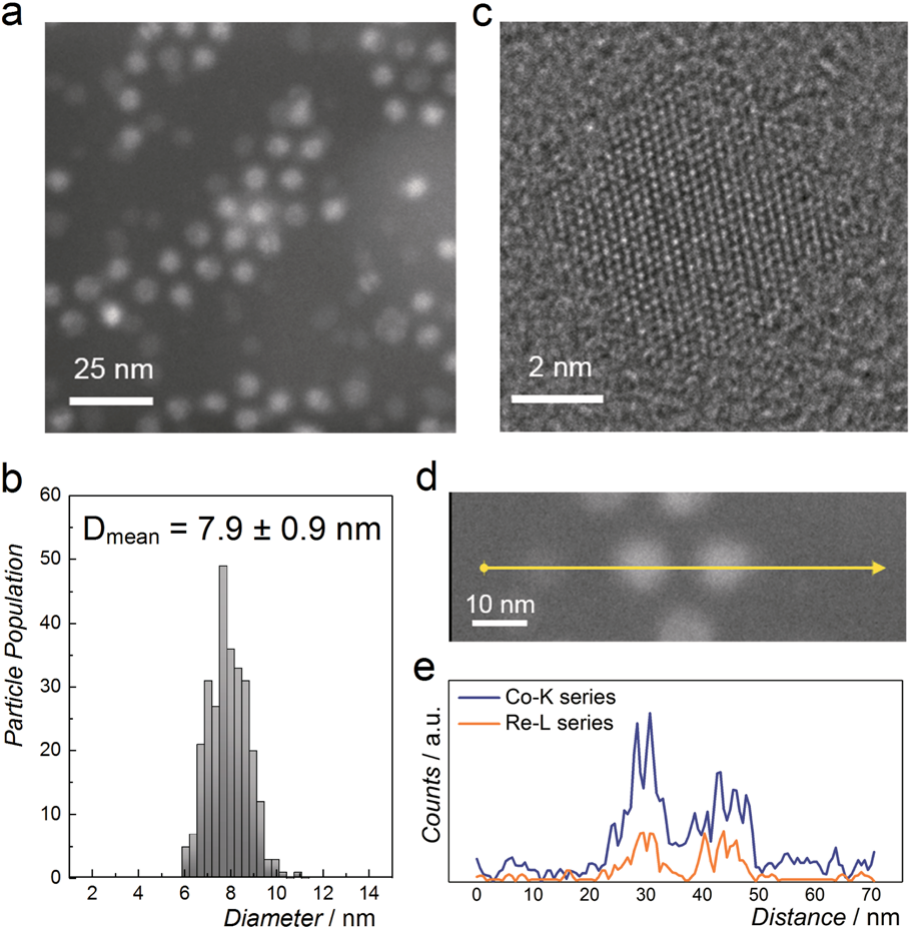
(a) HAADF-STEM image and (b) particle size distribution of NPs with nominal composition Co_0.85_Re_0.15_. (c) HRTEM image of a single particle. (d) HAADF-STEM image; and (e) its corresponding STEM-EDX line scan profile recorded along the arrow direction.

Compositional tuning of the Co_1−*x*_Re_*x*_ (*x* < 0.15) NPs is achieved by adjusting the relative molar ratio between the two metal carbonyl precursors. Rietveld refinements of the SR-PXRD patterns presented in Fig. S3† of NPs with varying Re-content give no indication for the existence of any crystalline phases with a high Re-content; we only identify Bragg reflections originating from the cubic β-Mn-type structure. Furthermore, a smooth expansion of the *a*-axis with increasing Re content for *x* < 0.15 is observed (Fig. 3), in good agreement with what is expected from Vegard's law approximation (dashed line).^16^ However, we were unable to obtain any uniform, single phased Co_1−*x*_Re_*x*_ NPs for *x* > 0.15. Attempts to synthesize such particles under our reaction conditions always resulted in a product with an estimated Re content of *ca.* 15 at% (from SR-PXRD). For example, the sample with nominal composition Co_0.60_Re_0.40_ gave *a* = 0.6223(1) nm (Fig. S3c†), corresponding to an estimated Re incorporation of 14.3 at% (Fig. 3). While no other crystalline phases were detected by SR-PXRD, complementary TEM investigations of Co_0.60_Re_0.40_ (Fig. S4 and S5†) reveal that the sample is highly heterogeneous with respect to particle size and chemical composition of the individual particles. Our findings clearly suggest that within the explored experimental window, the solubility limit of Re into single-phased β-Mn-type Co NPs is *ca.* 15 at%.

**Fig. 3:**
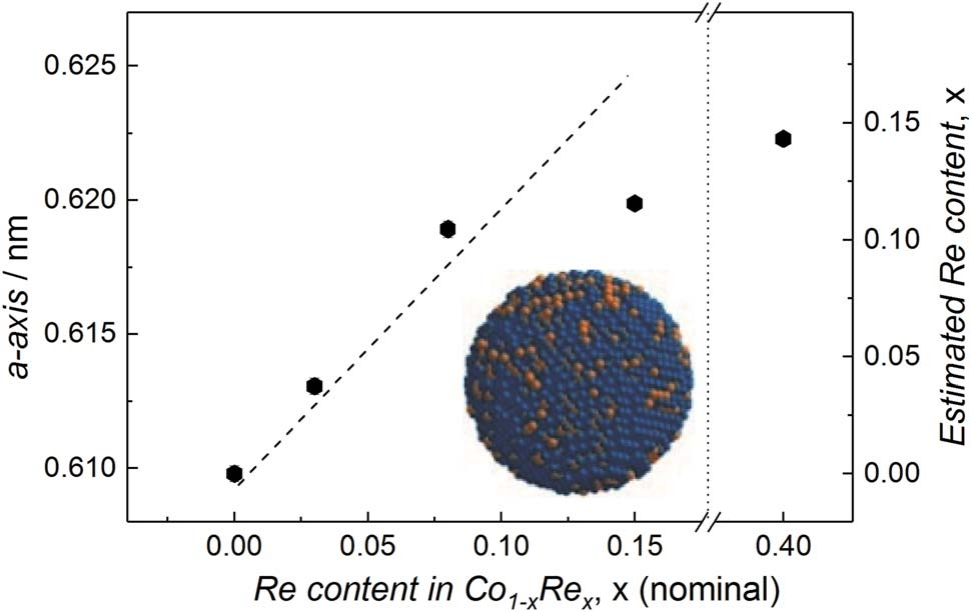
Refined *a*-axis *versus* nominal Re content for Co_1−*x*_Re_*x*_ NPs. Dashed line represents Vegard's law linear relation. Estimated Re content (based on Vegard's law) included on the right *y*-axis.

Returning to the SR-PXRD patterns of Co_1−*x*_Re_*x*_ (*x* < 0.15), we visually note that for similarly sized NPs the peak broadening becomes less extensive for partially Re-substituted NPs relative to the monometallic Co NPs (β-Mn-type structure).^9*a*^ We take this observation as an indication of stabilization of the β-Mn-type structure relative to the hcp/ccp polytypes by Re incorporation. To verify this hypothesis, DFT simulations were performed. In line with literature,^7^ DFT shows that the hcp polymorph represents the ground state for bulk Co (Fig. S6†). This is also the case for 2.5 nm Co NPs, with the β-Mn and the ccp polymorphs being 9 and 30 meV higher in energy than the hcp polymorph (note, according to Kitakami *et al.*,^17^ the ccp modification is stabilized relative to the hcp variant for Co NP < 20 nm, which is the reverse of what is the case for bulk Co). This situation becomes reversed for Co_1−*x*_Re_*x*_ NPs. For Co_0.85_Re_0.15_ NPs, the β-Mn polymorph is now calculated as the stable configuration with hcp and ccp respectively 68 and 91 meV higher in energy (see Fig. S6†). We propose that the less dense β-Mn structure is stabilized when a second element (here Re) is incorporated with site preference to the β-Mn lattice.

As catalytic application requires usage of the particles at elevated temperatures, the thermal stability of the β-Mn-type NPs with nominal composition Co_0.85_Re_0.15_ is studied using *in situ* SR-PXRD. Fig. 4a shows a contour plot of the diffraction patterns obtained on heating from 25 to 400 °C in diluted H_2_. Selected 2D-patterns are presented in Fig. 4b. Upon heating, all Bragg reflections of the β-Mn-type phase slightly shift to lower angles due to thermal expansion (see *e.g.* the blue and cyan diffractograms at 177 and 250 °C, Fig. 4b). For the β-Mn-type Co–Re NPs, the calculated linear thermal expansion coefficient is 25 × 10^−6^ °C^−1^ between 100 and 250 °C. Upon further heating, the NPs transform into the hcp phase at around 300 °C (green and red diffractograms in Fig. 4b). This phase transformation is in good agreement with reports on Co NPs.^14,18^ The sharp Bragg reflections of the hcp-type phase (red diffractogram at 350 °C, Fig. 4b) clearly show that a complete recrystallization and particle growth process has taken place, with an estimated change from 5 to 30 nm sized crystallites (using the Scherrer equation) for the β-Mn and hcp-type phases, respectively. The calculated unit cell dimensions of the hcp-type phase at 350 °C are *a* = 0.2516 nm and *c* = 0.4095 nm. When using the thermal expansion coefficients of hcp Co (linear coefficients along the a and *c* axes; 12.5 × 10^−6^ °C^−1^ and 17.8 × 10^−6^ °C^−1^, respectively),^19^ these values translate into a unit cell volume of 0.01105 nm^3^ per Co at 25 °C, which is very close to the value reported for pure hcp Co (0.01106 nm^3^ per Co).^20^ The high-temperature SR-PXRD data hence show that a drastic Re segregation occurs; the β-Mn-type Co–Re NPs transform into pure hcp Co, and consequently into a separate high-Re-content phase with too small particle size or too poor crystallinity to be detected by the applied method.

**Fig. 4:**
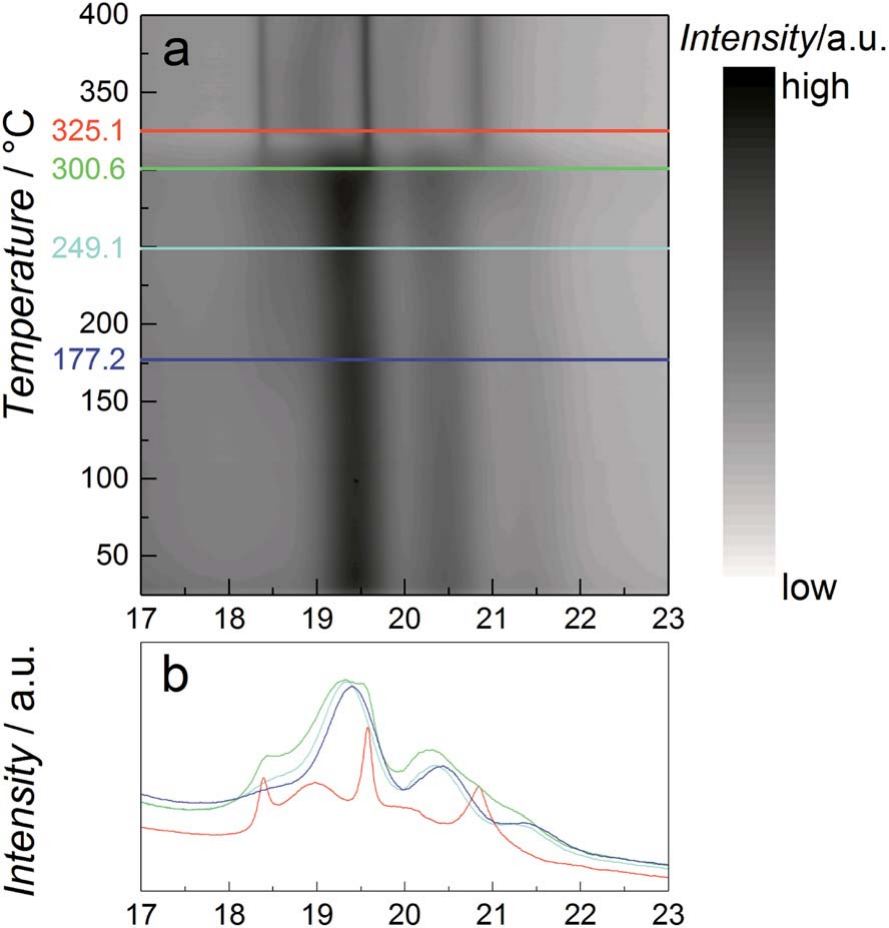
*In situ* synchrotron high temperature (a) PXRD contour plot of NPs with nominal composition Co_0.85_Re_0.15_ upon heating in 4 vol% H_2_/He atmosphere from 25 to 400 °C at a heating rate of 2 °C min^−1^. Colored lines correspond to selected PXRD-2D patterns presented in (b), *λ* = 0.06957 nm.

In summary, we report on the first synthesis of crystalline and monodisperse Co–Re NPs. According to Rietveld refinement, these NPs have the β-Mn-type structure with a distinct site preference of Re atoms in the 12-fold site. At the nanoscale, we find that the β-Mn-type structure is stabilized when Re is incorporated into the material. Homogeneous elemental distribution of Co and Re in the individual NPs was further demonstrated using HRTEM and STEM-EDX line scans. The synthesized Co–Re NPs form a solid solution up to a maximum of *ca.* 15 at% Re. In reducing atmosphere, the β-Mn polymorph is stable up to 300 °C. This defines the maximum operational temperature for this type of Co–Re NPs, a parameter of immense importance when being utilized as nano-catalysts for real world applications.

As the β-Mn-type Co–Re NPs are currently synthesized for the first time, their catalytic potential has not yet been explored. Although Co-containing Fischer–Tropsch catalysts are based on the hexagonal/cubic close-packed (hcp/ccp) Co polymorphs,^4*e*,21^ currently no routes for controlled hcp/ccp Co–Re NPs synthesis exist. Therefore, the synthesis of β-Mn-type Co–Re NPs represents a promising possibility to obtain well-defined model catalysts.^13^ These will be highly attractive for detailed investigations to elucidate the role of the Re promoter in the Co-based Fischer–Tropsch process. The β-Mn-type Co–Re NPs can be studied as such, or thermally converted into a hcp/ccp structure, which, although still very challenging, ought to be possible.

## Conflicts of interest

There are no conflicts to declare.

## Supplementary Material

NA-002-D0NA00097C-s001
