# Supplementary material for: One-pot synthesis of cobalt–rhenium nanoparticles taking the unusual β-Mn type structure
Source: Nanoscale Adv. 2020 Apr 7;2(5):1850–3. doi: 10.1039/d0na00097c (PMC9417266; doi:10.1039/d0na00097c)
Supplement: NA-002-D0NA00097C-s001 [file NA-002-D0NA00097C-s001.pdf]

Electronic Supplementary Material (ESI) for Nanoscale Advances.
This journal is © The Royal Society of Chemistry 2020

Supporting Information

One-pot synthesis of cobalt–rhenium nanoparticles taking the unusual β-Mn type structure

Eirini Zacharaki, G. Marien Bremmer, Ponniah Vajeeston, Maria Kalyva, Patricia J. Kooyman, Helmer Fjellvåg, and Anja O. Sjåstad*

Table of Contents

Experimental Procedures….. 2

Results and Discussion…..………………...…………………………………………..........5

References………………………………………………………………………………… 10

Experimental Procedures

Synthesis of cobalt–rhenium nanoparticles (NPs)

Co–Re NPs were synthesized by thermolysis of dicobalt octacarbonyl [$Co_2(CO)_8$, ≥ 90%] and dirhenium decacarbonyl [$Re_2(CO)_{10}$, 98%] in ortho-dichlorobenzene (*o*-DCB, 99%, anhydrous) with oleic acid (OA, ≥ 99%) as stabilizing agent. All chemicals were purchased from Sigma-Aldrich and used without further purification. In a typical $Co_{0.85}Re_{0.15}$ NP synthesis, 0.20 mmol $Re_2(CO)_{10}$ and 65 μL OA (0.205 mmol) were dissolved in 15 mL *o*-DCB under Ar flow. The solution was subsequently heated to 177 ± 1°C under stirring. In the meantime, 1.11 mmol $Co_2(CO)_8$ was dissolved in 3 mL *o*-DCB in a glove box (O_2 and H_2O levels <1 ppm). When the *o*-DCB/OA/$Re_2(CO)_{10}$ mixture reached the targeted temperature, the $Co_2(CO)_8$ precursor solution was rapidly injected into the hot mixture. The formed colloidal suspension was aged for 2–4 hours and subsequently quenched with 10 mL *o*-DCB. The NPs were flocculated using excess 2-propanol and isolated by centrifugation. After discarding the supernatant, the NP precipitate was washed with 2-propanol for at least three times before redispersion in hexane. In order to tune the Co–Re metal composition, the relative amounts of Co and Re carbonyl precursors were systematically adjusted (Table S1).

Table S1. Reactant quantities for the synthesis of $Co_{1-x}Re_x$ NPs.

	$Co_2(CO)_8$		$Re_2(CO)_{10}$		*o*-DCB	OA
Sample	(mg)	(mmol)	(mg)	(mmol)	(mL)	(mmol)
$Co_{0.97}Re_{0.03}$	440	1.286	28	0.043	18	0.205
$Co_{0.92}Re_{0.08}$	420	1.228	72	0.110	18	0.205
$Co_{0.85}Re_{0.15}$	380	1.111	132	0.202	18	0.205
$Co_{0.60}Re_{0.40}$	270	0.789	354	0.543	18	0.205

Characterization

The crystal structure and thermal stability of Co–Re NPs were investigated by synchrotron powder X-ray diffraction (SR-PXRD). All experiments were performed at the Swiss-Norwegian Beamlines (SNBL), station BM01A, at the European Synchrotron Radiation Facility (ESRF). Diffraction profiles were collected using a PILATUS 2M detector. Wavelength (λ = 0.06957 nm) was calibrated by means of a NIST Si standard. A PyFAI-based tool[1] was used for data reduction. For structural investigations, dried samples were loaded (inside a glove box, O_2 and

H_2O levels <1 ppm) in 0.7 mm diameter glass capillaries and sealed. Sets of diffraction patterns were collected at room temperature and the intensities were averaged to improve statistics. Rietveld refinements were performed using the Fullprof suite of programs[2] (2θ range 10–37°; Δ2θ = 0.0125°; 30 Bragg reflections, 10–13 variable parameters; manual background correction; typical Bragg R_F factor = 4.3, R_p = 10.4). For the temperature-dependent SR-PXRD experiments, an in-house-made quartz-capillary-based in situ cell was used. Sample heating was provided by the standard vertical hot air blower at the beam line. Flowing gas (≈5 mL min^{-1} 4 vol. % H_2/He (Messer Schweiz AG, 99.2%)) was supplied by a set of mass flow controllers calibrated for relatively small flows. The sample was heated in situ over the temperature range 25–400 °C at a rate of 2 °C min^{-1}. The diffraction patterns were recorded simultaneously using a collection rate of 4 images min^{-1}.

High-resolution transmission electron microscopy (HRTEM) was performed using a Cs-corrected FEI Titan Cubed microscope operating at 300 kV, equipped with a Gatan US1000 2k x 2k CCD, a Direct Electron DE-12 4k x 3k CCD and a high-angle annular dark-field (HAADF) detector, or with a monochromated FEI Tecnai F20ST/STEM operating at 200 kV, equipped with a Gatan US4000 4k x 4x CCD. Both microscopes were equipped with an energy dispersive X-ray detector (Oxford Instruments, X-MAX^N 100TLE Windowless) for EDX spectroscopy. Analysis of (STEM-) EDX data was done using AZTec software, version 3.1. All samples were prepared by drop casting 10 μL of the relevant NP dispersion onto ultrathin carbon film (< 3 nm) supported by lacey carbon film on 400 mesh copper grids (Ted Pella Inc.) and dried under inert atmosphere.

Computational details

All calculations were performed within the periodic density functional theory framework, as it is implemented in the VASP code.[3,4] The interactions between the core (Co:[Ar], and Re:[Xe]) and the valence electrons were described using the projector-augmented wave (PAW) method.[5,6] We have used the Perdew, Burke, and Ernzerhof (PBE)[7] spin polarized gradient corrected functional for the exchange–correlation part of the potential for the structural optimization. Our previous calculations suggested that structural parameters in metals can be reliably predicted only by using large energy-cutoff to guarantee basis-set completeness. Hence, we have used a cut-off of 700 eV. The atoms were deemed to be relaxed when all atomic forces were less than 0.02 eV $Å^{-1}$ and the geometries were assumed to be optimized when the total energy converged to less than 1 meV between two consecutive geometric optimization steps. Only the FM states were considered for all phases studied.

Different sizes of the nanoparticles (NP) have been constructed from optimized bulk phase structures through different supercell sizes. The **k**-points were generated using the Monkhorst-Pack method with a grid size of $1\times1\times1$, for structural optimization for NPs. For the 15% Re substituted Co initial structure generation, the structural data for Co in $P6_3/mmc$, Fd-3m, $P4_132$ were taken from the ICSD database and we used the *ab initio* random searching structure (AIRSS)[8] method to generate possible model structures for the Re substitution in Co matrix, coupled with VASP calculations. Iterative relaxation of atomic positions was stopped when the change in total energy between successive steps was < 1 meV/cell.

Results and Discussion

HRTEM-EDX elemental analysis of $Co_{1-x}Re_x$ NPs.

Table S2. Metal composition of $Co_{1-x}Re_x$ bimetallic NPs.

Sample	At. % Re[a]
$Co_{0.97}Re_{0.03}$	ND
$Co_{0.92}Re_{0.08}$	5.1 ± 0.9
$Co_{0.85}Re_{0.15}$	12.2 ± 1.3

[a] Averaged data from single nanoparticle (randomly selected) HRTEM-EDX analysis. "ND" not determined.

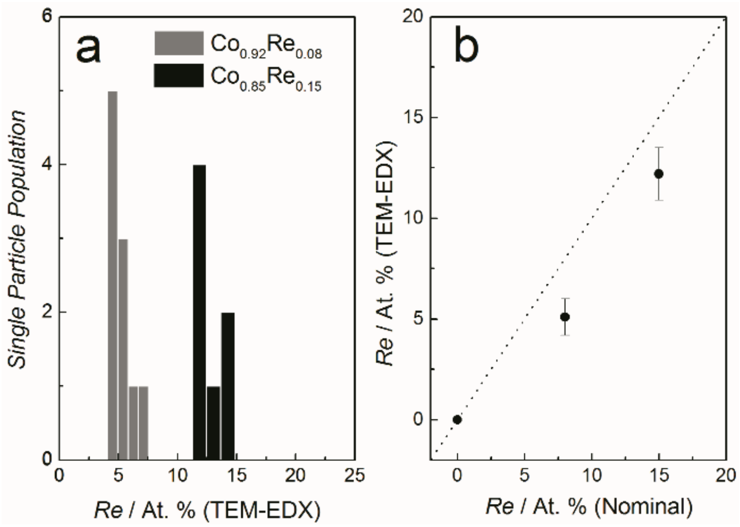

Figure S1. a) Metal composition determined from HRTEM-EDX of bimetallic Co_{1-x} Re_x NPs on single nanoparticles. b) Estimated composition determined by HRTEM-EDX versus nominal composition.

Structural parameters determined by Rietveld refinement.

Table S3. Crystallographic data[a] of *β*-Mn-type Co–Re NPs with nominal composition $Co_{0.85}Re_{0.15}$.

Atom	Site	x	Y	z	Occupancy
**Co1** x,x,x	1 (8*c*)	0.0634(5)	0.0634(5)	0.0634(5)	1.000
**Re1** x,x,x	1 (8*c*)	0.0634(5)	0.0634(5)	0.0634(5)	0.000
**Co2** 1/8,y,z	2 (12*d*)	0.125	0.193(2)	0.461(1)	0.818(7)
**Re2** 1/8,y,z	2 (12*d*)	0.125	0.193(2)	0.461(1)	0.182(7)

[a] T = 20 °C; R_{wp} = 10.1%; Space group: $P4_132$; Unit cell parameter, *a* = 0.6199(1) nm.

Site Preferences in *β*-Mn-type Co–Re phases.

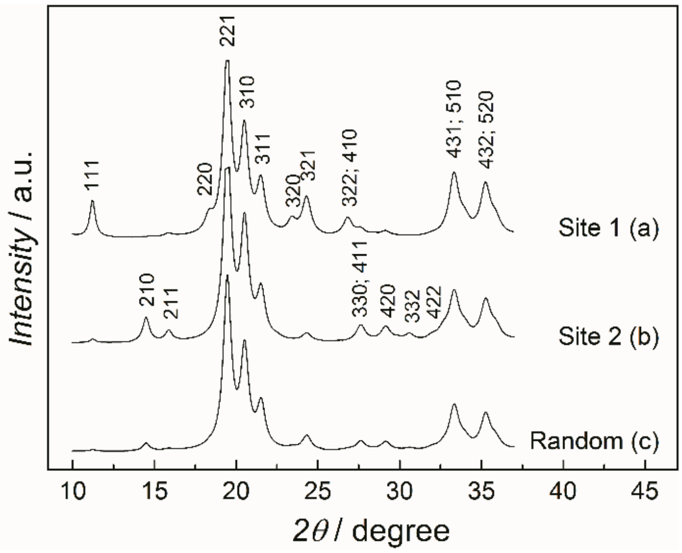

Figure S2. Calculated X-ray diffraction patterns of *β*-Mn-type Co–Re (Re, 1/6 fraction). In the simulated patterns, all Re atoms are assumed to occupy only a) site 1 (8*c*); b) site 2 (12*d*); or c) are randomly distributed in the *β*-Mn-type Co lattice. Taking the intensities of the 111, 210 and 211 Bragg-reflection into consideration, the diffraction patterns of the $Co_{1-x}Re_x$ for x < 0.15 NPs (Figure 1 in main text and Figure S3) indicate that almost all Re atoms occupy site 2 (12*d*).

Compositional variation of the $Co_{1-x}Re_x$ (x < 0.15) NPs.

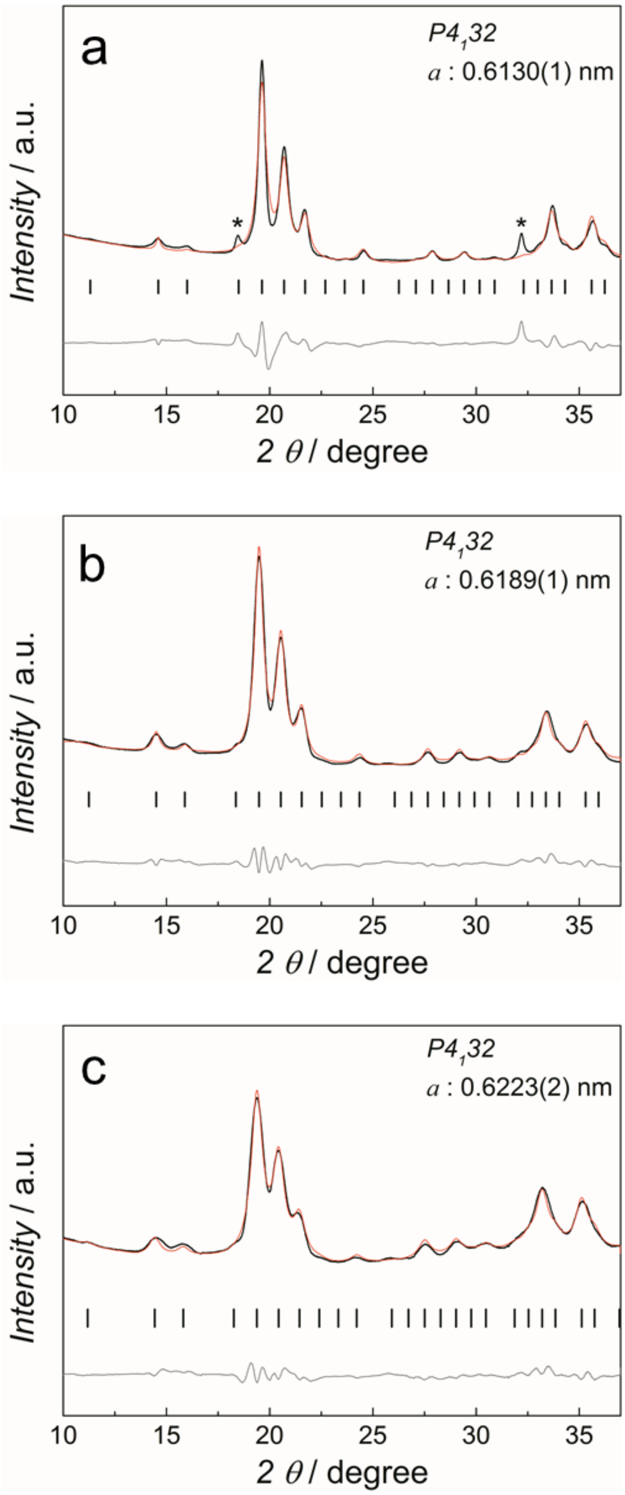

Figure S3. Rietveld refinements of synchrotron PXRD intensity profiles of a) $Co_{0.97}Re_{0.03}$; b) $Co_{0.92}Re_{0.08}$; and c) $Co_{0.60}Re_{0.40}$ NPs. Observed (black), calculated (red) and difference (gray) profiles are shown along with the positions for Bragg reflections (vertical bars). Impurity denoted with asterisk (*****). λ = 0.06957 nm.

For the $Co_{0.97}Re_{0.03}$ sample, SR-PXRD revealed some weak additional reflections (indicated by asterisk in Figure S3a). The origin of these reflections is not fully understood; however, possibly some can be related to hcp/ccp intergrowth particles.[9] With increasing Re content (Figure S3b–c) the above mentioned reflections disappear, yielding single-phase particles.

TEM imaging of $Co_{0.60}Re_{0.40}$ sample.

Representative TEM images of the $Co_{0.60}Re_{0.40}$ NPs are presented in Figure S4. Particles are characterized by a tri-modal size distribution; the main population being spherical bimetallic Co–Re NPs with an average diameter of ca. 7 nm. Some ultra-small clusters and larger aggregates (with sizes of ca. 1 nm and 50–70 nm, respectively) were also identified. STEM-EDX mapping suggests that the latter are Re-rich (Figure S5).

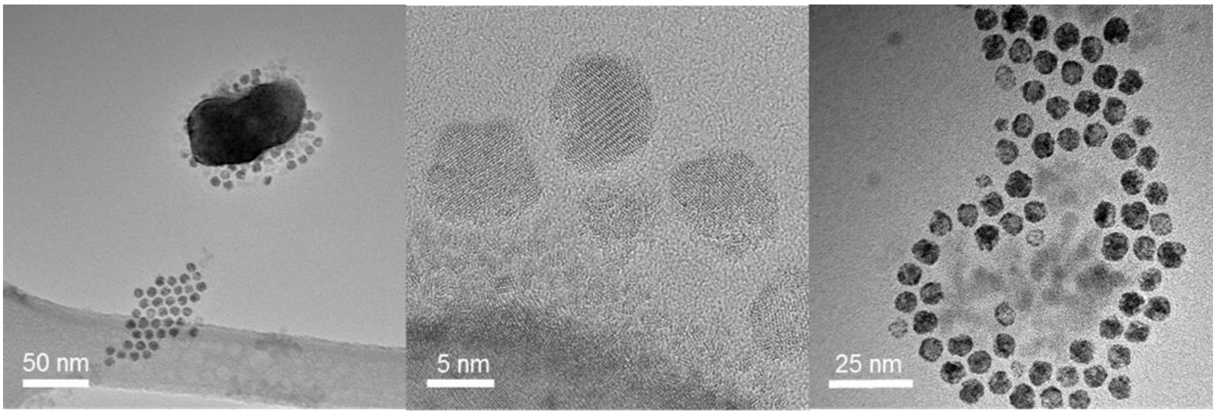

Figure S4. Representative TEM images of $Co_{0.60}Re_{0.40}$.

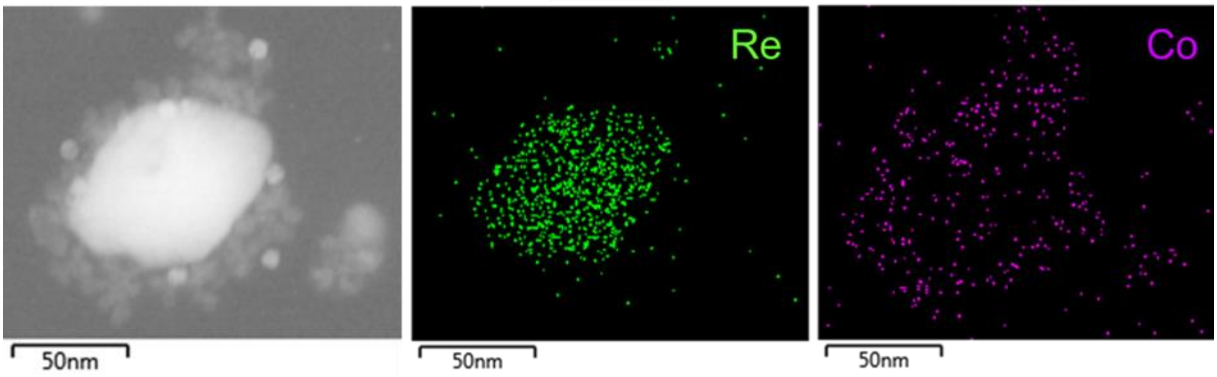

Figure S5. HAADF-STEM image and EDS mapping of Re-L (green) and Co-K (purple) of $Co_{0.60}Re_{0.40}$.

DFT simulations.

The lowest energy configurations of different Co polymorph derived NPs and their corresponding 15% Re substituted Co NPs are shown in Figure S6 along with their relative stability.

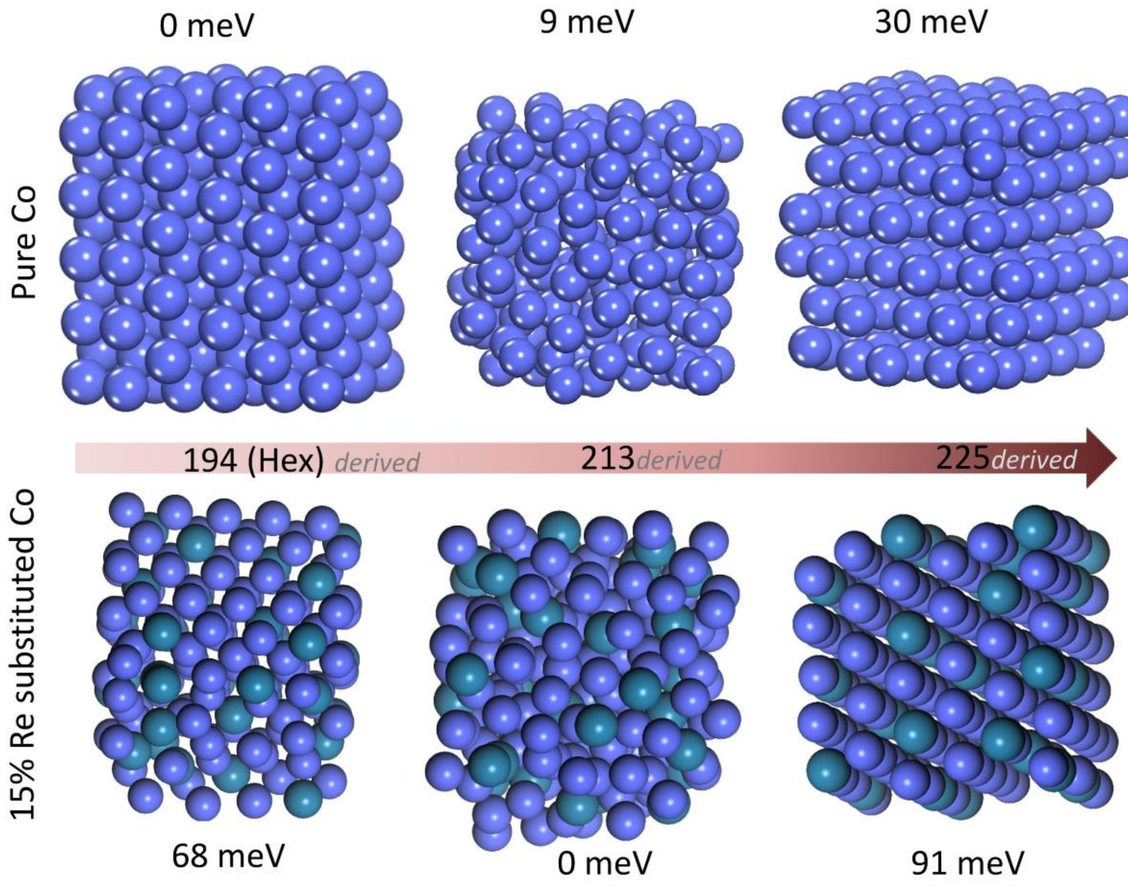

Figure S6 Relative stability of optimized nanoparticles (dimension of ca. 2.5 nm) of pure Co (upper) and $Co_{0.85}Re_{0.15}$ (lower) of the hcp (left), β-Mn type (center) and ccp (right) polymorphs.

References

1 V. Dyadkin, P. Pattison, V. Dmitriev, and D. Chernyshov, *J. Synchrotron Radiat.,* 2016, **23**, 825-829.

2 J. Rodríguez-Carvajal, *Phys B,*1993, **192**, 55-69.

3 G. Kresse and J. Furthmüller, *Phys. Rev. B,* 1996, **54**, 11169-11186.

4 G. Kresse and J. Furthmüller, *Comput. Mater. Sci.,* 1996**, 6**, 15-50.

5 P.E. Blöchl, *Physical Review B,* 1994, **50**, 17953-17979.

6 G. Kresse and D. Joubert, *Physical Review B,* 1999, **59**, 1758-1775.

7 J. P. Perdew, K. Burke, and M. Ernzerhof, *Phys. Rev. Lett.,* 1996, **77**, 3865-3868.

8 J. P. Chris and R. J. Needs, *Journal of Physics: Condensed Matter,* 2011, **23**, 053201.

9 E. Zacharaki, M. Kalyva, H. Fjellvåg, A. O. Sjåstad, *Chem. Cent. J.,* 2016, **10**, 10.
